# Protocol for short-term *in vitro* propagation of patient-derived group 4 medulloblastoma MBT375 tumor cells

**DOI:** 10.1016/j.xpro.2026.104809

**Published:** 2026-08-27

**Authors:** Stefan Custers, Chitra Venugopal, Sheila K. Singh

**Affiliations:** 1 Centre for Discovery in Cancer Research, McMaster University, 1280 Main St W, Hamilton, ON L8S 4L8, Canada; 2 Department of Surgery, McMaster University, 1280 Main St W, Hamilton, ON L8S 4L8, Canada; 3 School of Cancer and Pharmaceutical Sciences, Comprehensive Cancer Centre, King’s College London, London, UK

**Keywords:** Cell Biology, Cell culture, Cancer, Model Organisms

## Abstract

Durable *in vitro* cell culturing is an urgent unmet need for the pre-clinical development of novel group 4 medulloblastoma (G4-MB) specific therapies. Here, we present a protocol for the establishment and propagation of G4-MB tumor cells for short-term *in vitro* culturing. We describe brain tumor cell isolation, murine intracranial engraftment, *in vitro* culturing. and assay seeding. Our approach enables short-term *in vitro* passaging and drug screening. For long-term propagation of these G4-MB models, serial engraftment is required.

For complete details on the use and execution of this protocol, please refer to Custers et al.^1^

## Highlights

• Detailed instructions how to isolate and maintain group 4 medulloblastoma cells
• Guidance on maintaining healthy and stable cell populations *in vitro*
• Workflow for efficient use of patient-derived group 4 medulloblastoma cells in assays

## Abstract (editor highlights)

Publisher’s note: Undertaking any experimental protocol requires adherence to local institutional guidelines for laboratory safety and ethics.

## Graphical abstract

**Figure:**
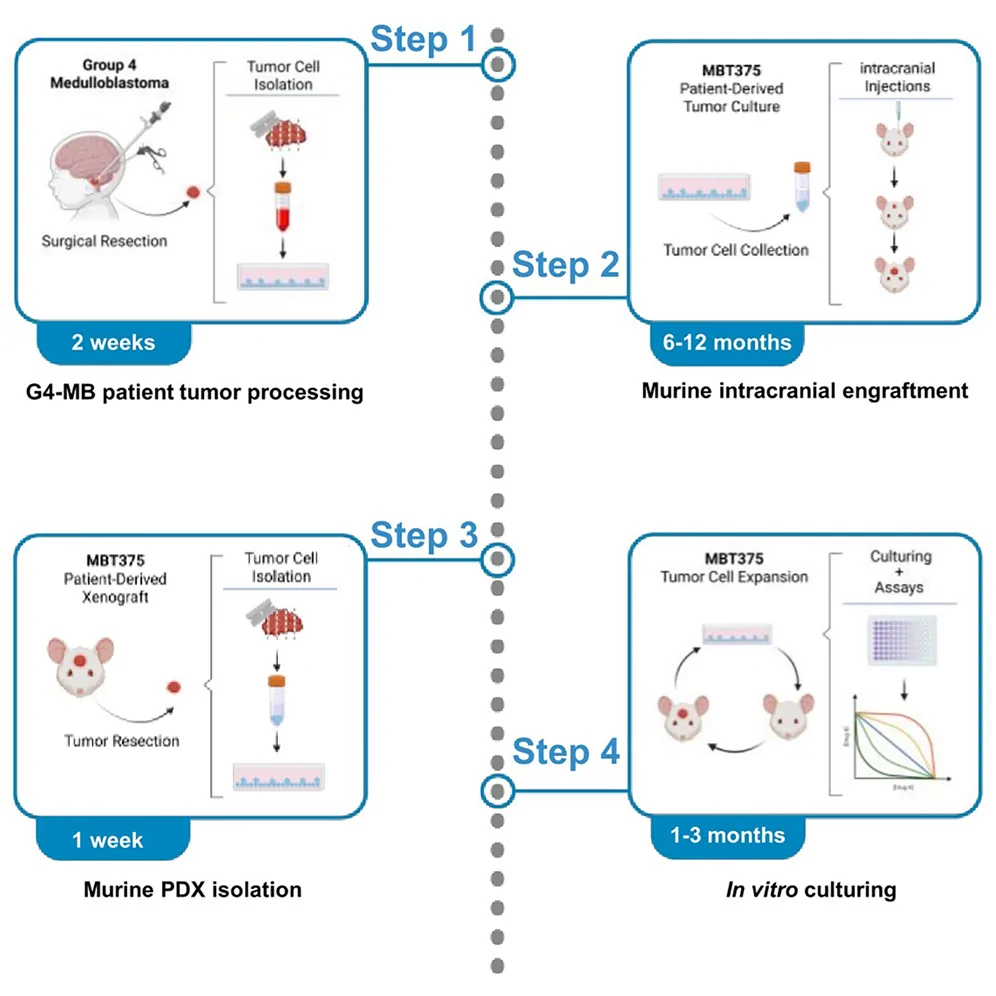

## Teaser

Durable *in vitro* cell culturing is an urgent unmet need for pre-clinical development of novel group 4 medulloblastoma (G4-MB) specific therapies. Here, we present a protocol for the establishment and propagation of G4-MB tumor cells for short-term *in vitro* culturing. We describe brain tumor cell isolation, murine intracranial engraftment, *in vitro* culturing. and assay seeding. Our approach enables short-term *in vitro* passaging and drug screening. For long-term propagation of these G4MB models, serial engraftment is required.

## Before you begin

### Background

Group 4 medulloblastoma (G4-MB) patient-derived tumor cells are notoriously difficult to maintain *in vitro.* The lack of *in vitro* models has severely limited pre-clinical advancement of G4-MB research.^2^ To address these limitations, we devised a method of isolating and culturing patient-derived G4-MB tumors. By using this protocol, we successfully established two *in vitro* G4-MB lines; MBT375 and MBT417, which can be propagated for a short period of time. Here, the MBT375 line is used for demonstration purposes.^1^

### MBT375 tumor phenotypes

**Figure 1:**
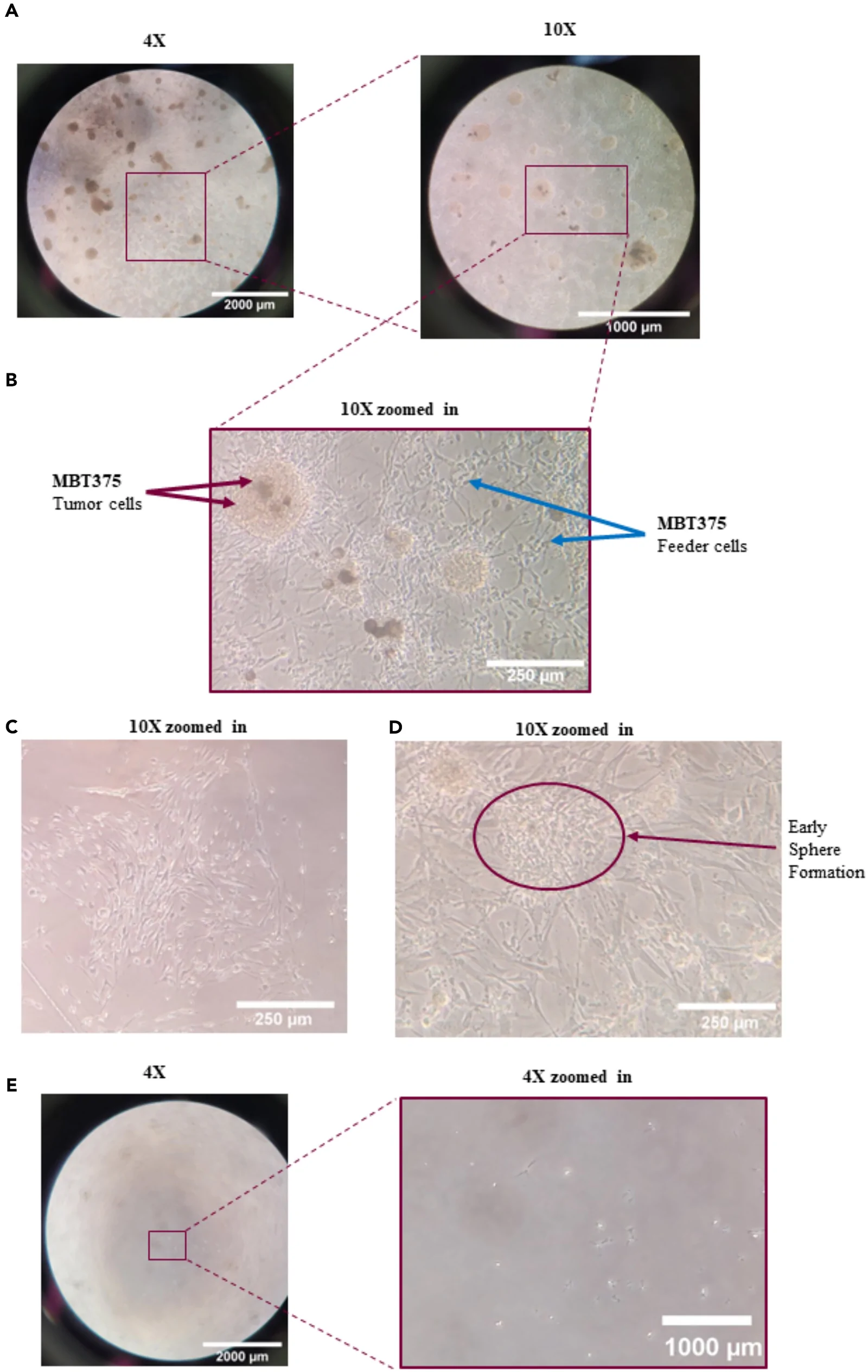
Phenotypical characteristics of MBT375 *in vitro* cultures (A) Microscope Image of a healthy and stable culture of MBT375 displaying an adherent monolayer of feeder cells, with tethered and suspension tumor spheres at 4× and 10× magnification. (B) Magnification of panel (A), highlighting MBT375 tumor spheres (red) and feeder cells (blue). (C) Feeder and tumor cell integration site, image taking after tumor spheres were removed the rinsing. (D) Feeder monolayer, with a feeder-tumor integration site starting to form a new tumor sphere (purple circle). (E) Microscope image of left over adherent single cells of MBT375 cells, indicative of the unique phenotypic properties of adherent MBT375 cells at 4× magnification. Images were taken with a camera through a light microscope lens. Scale bars: 4X = 2000 m, 4X zoomed = 1000 m, 10X = 1000 m, 10X zoomed in 250 m.

The main limiting factor for *in vitro* propagation of G4-MB cells is their low proliferative capacity. The use of Poly-L-Ornithine + Laminin (POL) coating is an essential mitigating strategy for this limitation. The use of POL-coated plates generates two phenotypically distinct cell populations: MBT375 tumor cells with a small and smoothly rounded cell body and adherent MBT375 feeder cells with large and elongated cell bodies. On POL, MBT375 feeder cells provide a supportive niche which greatly improves *in vitro* durability. This adherent layer supports the growth of tumor cell spheres which exist both tethered to the monolayer and in suspension (Figures 1A and 1B). MBT375 tumor and feeder cells co-exist adherently on POL as localized patches of integrated sites, as depicted in Figure 1C, where both phenotypes can be appreciated clearly. These are areas where spheres tether to the monolayer and where new spheres are formed (Figure 1D). When viewed under a light microscope, MBT375 tumor cells appear as small bright dots in suspension. Adherent MBT375 tumor cells have a small bright cell body and display two to three fine protrusions extending outwards from their main cell body (Figure 1E). An important note is that stressed MBT375 tumor cells (after passaging, thawing or isolation from mouse brains) have an asymmetrical rough-edge and debris-like phenotype that can easily be mistaken for dead cells or debris. Due to the low proliferative capacity of G4-MB cultures, serial engraftment in mouse brains remains the main method for extended bulk tumor cell propagation.

This protocol is structured in four main parts (1) isolation of G4-MB cells from a freshly resected patient tumor, (2) intracranial engraftment of MBT375 in mice for bulk cell expansion, (3) tumor cell isolation form mouse brains at endpoint, (4) *in vitro* culturing of MBT375 cells, including passaging, splitting, seeding and a proposed workflow from patient tumor to assays for optimal usage of *in vitro* cultured tumor cell populations.

### Innovation

Currently there is a limited number of permanent G4-MB in vitro cell lines available, however they lack intratumoral heterogeneity, often display genetic/phenotypic drift and their subgroup affiliations are contested in literature. Durable patient-derived *in vitro* G4-MB models are non-existent.^2^^,^^3^^,^^4^ The lack of suitable pre-clinical and discovery models is the main bottleneck for development of better therapies for G4-MB patients. An important novelty of this model is the ability to circumvent current limitations of G4-MB in vitro culturing properties, which enables the short-term *in vitro* culturing of patient-derived G4-MB tumor cells.

### Institutional permissions (if applicable)

Human G4-MB resected tumors were obtained through the McMaster Department of Surgery. Informed consent was obtained from all G4-MB patients prior to tumor cell isolation. All *in vivo* studies were performed according to, and approved by, the McMaster University Animal Research Ethics Board (AREB) approved protocols.

Experimenters following this protocol must gain permission from and abide by their local relevant authorities.

### Preparation of NeuroCult complete medium

**Timing: ∼10 min**

Preparation of the main cell culturing medium used for this model.

1. Prepare 500 mL of NCC medium by supplementing NeuroCult NS-A Basal media with NeuroCult NS-A Proliferation Supplement (50 mL), 500 μl of 0.2% Heparin Solution, 1% antibiotic-antimycotic solution, epidermal growth factor (20 ng/mL) and fibroblast growth factor (10 ng/mL) (Table 1).

**Table 1:** Composition of NCC growth medium

Reagent	Company	Cat#
NeuroCult NS-A Basal Medium (human)	STEMCELL technologies	05750
NeuroCult NS-A Proliferation Kit	STEMCELL technologies	05751
Heparin Solution	STEMCELL technologies	07980
hEGF	STEMCELL technologies	78006.2
hFGF	STEMCELL technologies	28003.2
Antibiotic-antimycotic solution	Wisent	450-115-EL
2. Store the NCC media at 4°C.
3. Prior to use, warm an appropriate volume of NCC media in a water bath at 37°C for 5 min.

***Note:*** Due to the low stability of growth factors (EGF and FGF), we recommend storing NCC bottles for a maximum of 2 weeks at 4°C.

### Preparation of Poly-L-Ornithine + Laminin coated plates

**Timing: 1 day**

Cell culturing plates are prepared with a coating in order to allow adherent cell culture.

4. Dilute Poly-L-Ornithine (0.1%) solution 1:5 in PBS.
5. Add appropriate volume to wells (3 mL per 6 wp well).

***Note:*** Poly-L-Ornithine is a hydrophobic solution that will be repelled from the edges of a plate’s wells. Make sure the solution’s volume is high enough to prevent this edge repelling effect as this severely reduce the quality and longevity of the POL coating.

6. Incubate for a minimum of 1–4 hour at 37°C (24 h is also possible).
7. Dilute 1 mg/mL Laminin (1:200) in PBS.
8. Remove Poly-L-Ornithine dilution and directly add 3 mL laminin to wells.

***Note:*** Laminin is a hydrophobic solution that will be repelled from the edges of the well. Make sure the volume is high enough to prevent this edge repelling effect as this will cause POL coating to gradually peel off the plate. Do not allow plates to dry once coated.

9. Incubate for 24 h at 37°C.
10. The plate is ready for use. Remove Laminin solution and perform 3 PBS washes prior to adding cells in NCC.
11. Plates can be kept in an incubator 37°C for 1 or 2 days, or stored at 4°C for 1 to 2 weeks. Wrap plates with parafilm when storing at 4°C.

***Note:*** Do not use plates that have dry spots or have completely dried out. Top up plates with PBS when necessary.

## Key resources table

**Table:** 

REAGENT or RESOURCE	SOURCE	IDENTIFIER
**Biological samples**		

Surgically Resected G4-MB tumor	McMaster Department of Surgery	N/A

**Chemicals, peptides, and recombinant proteins**		

NeuroCult NS-A Basal Medium (human)	STEMCELL technologies	05750
NeuroCult NS-A Proliferation Kit	STEMCELL technologies	05751
Heparin Solution	STEMCELL technologies	07980
hEGF	STEMCELL technologies	78006.2
hFGF	STEMCELL technologies	28003.2
Antibiotic-antimycotic solution (100×)	Wisent	450-115-EL
Lonza Walkersville MycoZap™ prophylactic	Lonza	VZA2031
Poly-L-Ornithine solution	Sigma	P4957-50mL
Laminin	BD Biosciences	354232
Sterile PBS, pH 7.4	Gibco	10010023
TrypLE	Gibco	12605010
DNAse I	Worthington	LK003170
Liberase Blendzyme 3	Millipore Sigma	5401119001
Ammonium Chloride Solution (RBC lysis buffer)	StemCell	7850
Trypan Blue	Gibco	15250061
Dimethyl sulfoxide (DMSO)	Sigma-Aldrich	D8418

**Experimental models: Organisms**		

NOD-SCID-IL2Rgcnull (NSG) mice (2–4weeks old female/male)	The Jackson Laboratory	JAX: #005557

**Other**		

TC-treated culture dishes, 10 cm	Corning	08-772-22
TC-treated multiple well plates, 6 well	Corning	07-200-80
TC-treated multiple well plates, 12 well	Corning	07-200-83
TC-treated multiple well plates, 96 well	Corning	07-200-90
Corning Costar Ultra-low attachment multiple well plate, 6 well	Corning	CLS3471
Corning Costar Ultra-low attachment multiple well plate, 24 well	Corning	CLS3473
Single-edge industrial razor blades	VWR	55411-050
16-gauge precision glide needle	BD Biosciences	305198
DB Disposable Syringes, 3mL	Thermo Fisher Scientific	B309657
Falcon Cell Strainer (70 μm, Nylon) for 50mL conical tubes	Corning	CLS352350
15 mL Falcon Tubes	Corning	CLS352095
50 mL Falcon Tubes	Corning	CLS352070
Eppendorf Safe-Lock micro test tubes, 1.5 mL	Merck	EP0030120086
Incubating rocking platform shaker	VWR	444-0763
Cell Countess	Thermo Fisher Scientific	ZGEXSCCOUNTESS2FL

**Software and algorithms**		

BioRender	Institute subscription	https://biorender.com

## Materials and equipment

**Table:** NeuroCult Complete media

Reagent	Final concentration	Amount
NeuroCult NS-A Basal Medium	N/A	500 mL
NeuroCult NS-A Proliferation kit	N/A	50 mL
0.2% Heparin Solution	N/A	0.5 mL
hEGF (100 ng/mL)	20 ng/mL	0.2 mL
hFGF (50 ng/mL)	10 ng/mL	0.2 mL
Antibiotic-antimycotic (100×)	1×	5 mL
**Total**	N/A	**555.9 mL**

[Store at 4C for up to 2 weeks]

**CRITICAL:** All components are added to the NeuroCult NS-A Basal Medium bottle. Mark bottle as “NCC Complete” to distinguish it from Basal Medium.

## Step-by-step method details

### G4-MB patient tumor processing

**Timing: 2 weeks**

In order to establish a patient-derived tumor cell line, tumor cells are extracted and isolated from surgically resected tumors. This section describes the steps performed when a freshly dissected tumor arrives in the lab for tumor cell isolation.

***Note:*** It is advised to cryo-preserve a considerable amount (10%) of freshly isolated tumor cells (passage zero, p0) stocks as they most closely represent the patient tumor.

1. Preparation.
  a. Pre-warm an incubating rocking platform shaker to 37°C.
  b. Thaw RBC lysis buffer in heat bath.
  c. Thaw a 200 μL Liberase aliquot (2.5 mg/mL) in heat bath.
  d. Pre-warm sterile 15 mL sterile PBS pH 7.4 in heat bath.
  e. Pre-warm NCC media in heat bath.
  f. Retrieve the resected tumor from the OR.

**CRITICAL:** Keep tumor on ice or at 4°C at all times prior to tumor processing.

**Figure 2:**
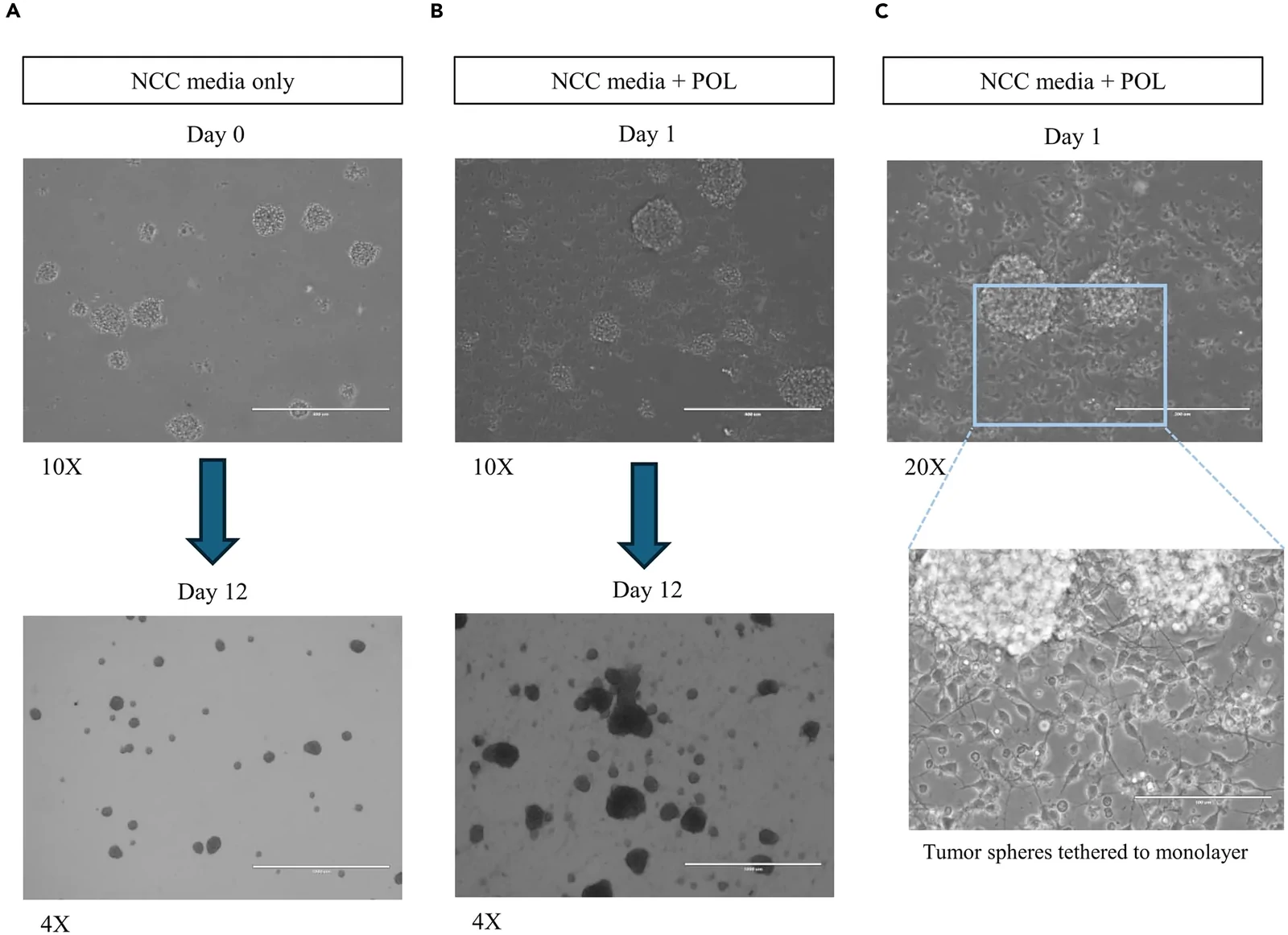
Culturing conditions of patient derived MBT375 tumor cells at isolation (A) Comparison of MBT375 suspension culture at day 0 and 12 in NCC cell culturing media. (B) Comparison of MBT375 culture at day 0 and 12 in NCC cell culturing media on Poly-L-Ornithine + Lamine (POL) coated plates. (C) Magnification of culture (B) highlighting tumor spheres tethered to the monolayer. Images were taken with an EVOS Cell Imaging System. Scale bars: 4X = 1000 µm, 10X = 400 µm, 20X = 200 µm, 40X = 100 µm.

***Note:*** Plate size is dependent on the number of viable cells. Determine plate size as such that the spheroids form a confluent surface.

2. Tumor assessment.
  a. Place tumor on a 10 cm plate inside a culturing biosafety hood.
  b. Take photographic images and document phenotype.
  c. Collect a representative 10% portion of the tumor tissue in an Eppendorf tube for cryo-preservation, and keep on ice.
3. Mechanically dissociate the tumor with razorblades until primary structure is reduced into a sludge.
4. Enzymatic tumor dissociation.
  a. Mix thawed Liberase aliquot with the pre-warm PBS aliquot.
  b. Use a 10 mL serological pipet and pipet gun to collect tumor sludge by rinsing the 10 cm plate twice with 7.5 mL Liberase PBS mix.
  c. Collect the tumor sludge in a 50 mL tube.
  d. Place 50 mL tube on the pre-heated incubating rocking platform shaker.
  e. Incubate for 15 minutes at 37°C at a medium rocking speed.
  f. Use the incubation time to snap-freeze and store the 10% representative tumor tissue portion.
5. Collection of tumor cells.
  a. Place a 70 μm nylon cell strainer on top of an opened 50 mL tube.
  b. Allow dissociated tumor sludge to sift through the filter (5 mL at a time).
    ***Note:*** Gently stirring with a 1 mL pipet tip helps speed up the sifting process.
  c. Rinse with 5 mL PBS to ensure all tumor cells have passed through the filter into the fresh 50 mL tube.
  d. Centrifuged the flow through for 5 min at 430 × *g.*
  e. Remove supernatant.
  f. Resuspend in 1 mL PBS.
6. Red Blood cell lysis.
  a. Assess the redness of cell pellet and resuspend with appropriate volume of RBC lysis buffer (max 10 mL).
  b. Incubate at room temperature (20°C–25°C) for 5 min.
  c. Dilute RBC lysis buffer with PBS at a 1:1 ratio.
  d. Centrifuge for 5 min at 430 × *g.*
  e. Remove supernatant.
7. Cell counting and plating. Troubleshooting 1.
  a. Resuspend cell pellet in appropriate volume of NCC (1–5 mL).
  b. mix 10 μL of resuspended cells with 10 μL Trypan Blue.
  c. Count cells with a Cell Countess.
  d. Cryo-preserve a subset of cells (depending on cell count) in NCC + 10% DMSO and store in liquid nitrogen for long-term storage.
    ***Note:*** We recommend cry-preserving at least 50% of cells, use 25% of cells for serial engraftment in mice, and use 25% of cells for immediate *in vitro* and molecular experimental needs. Depending on the tumor cell yield these percentages may change, depending on the researcher’s priorities.
  e. Dilute all remaining cells to 12 mL NCC, add 120 μL MycoZap and plate in 10 cm plate.
8. Incubate cells in an incubator for approximately 1 week and monitor sphere formation and media consumption.
9. When spheres have formed, the cells can be directly utilized for intracranial engraftment in mice and for plating on POL-coated plates for subsequent *in vitro* culturing (Figure 2).

10. For POL culturing, plate cells in low volume (7 mL NCC per 10 cm plate).
11. Incubate for 24–48 h to allow cells to adhere to the plate.
12. Gently top up media to 12 mL.
13. Incubate for approximately 1 week at 37°C until a stable culture has formed.

### Intracranial engraftment in mice

**Timing: 6–12 months**

Once a stable patient-derived tumor culture is established, cells are intracranially engrafted into immunocompromised NSG mice for bulk expansion, with a subset reserved for in vitro applications. The complete step-by-step procedure for preparing cells for intracranial injection is provided below.

***Note:*** Tumor-bearing mice typically reach endpoint after 9–12 months (in vivo passage 1, iP1). Subsequent serial engraftments shorten this period to 6–9 months. *In vitro* cells can be used in parallel with *in vivo* expansion. It is advised to cryo-preserve all tumor cells that are not allocated for engraftment or *in vitro* work at this stage.

14. Collect MBT375 cells (in suspension) into a falcon tube.
15. Rinse monolayer gently by pipetting 1 mL of PBS with a P1000 Pipette and collect in falcon tube.

***Note:*** Be gentle to prevent detachment of adherent feeder cells.

16. Add an appropriate volume of warm NCC media to MBT375 culturing plate and leave in incubator for further culturing.
17. Centrifuge the falcon tube containing the collected MBT375 cells 430 × *g*, 10 min.
18. Resuspend cell pellet in 500 μL TrypLE + 10 μL DNAse I.
19. Incubate at room temperature (20°C–25°C) and frequently resuspend with P200 to dissociate spheres until they are fully disassociated. This may take up to 5 min.

***Note:****A* P1000 pipette generates a higher sheer force while resuspending and causes cell death.

20. Observe cells under light a microscope until cells are fully dissociated into single cells.

***Note:*** MBT375 cells can be vulnerable to enzymatic dissociation induced cell death. Extracellular DNA accumulation results in sticky cell/DNA conglomerates which should be removed by pipetting immediately when observed.

21. Dilute single cells in TrypLE with 5 mL NCC.
22. Centrifuge cells 430 × *g*, 10 min.

**CRITICAL:** MBT375 tumor cells are very small in size. For this reason, we recommend all centrifugation steps to be performed at 430 × *g* for at least 10 min to ensure all single cells are pelleted.

23. Carefully discard supernatant by pipetting.
24. Resuspend cell pellet in 500 μL PBS (depending on cell pellet size).
25. Count cells in duplicate with Trypan Blue and Cell Counter Troubleshooting 1.
26. Adjust cell suspension to proper volume (500,000 cells per 5 μL).
27. Inject mice with at least 500,000–1,000,000 cells in 10 μL per mouse as per our published protocols^1^ Troubleshooting 2.

***Note:*** A minimum of 1,000,000 cells are preferred for initial engraftment. However, engraftment rates can vary between MBT375 cell populations. To ensure engraftment success rates and tumor growth, the injection of intact spheres after mechanical dissociation can be considered. When injecting spheres, the number of mice should be estimated by cell counts determined at the previous tumor cell isolation step.

### Mouse PDX tumor isolation

**Timing: 1 week**

Upon mouse Endpoints after approximately 9–12 months, tumor cells are harvested yielding up to retrieved up to 50 million cells per mouse brain. Below is a detailed description of the PDX tumor isolation procedure.

**Figure 3:**
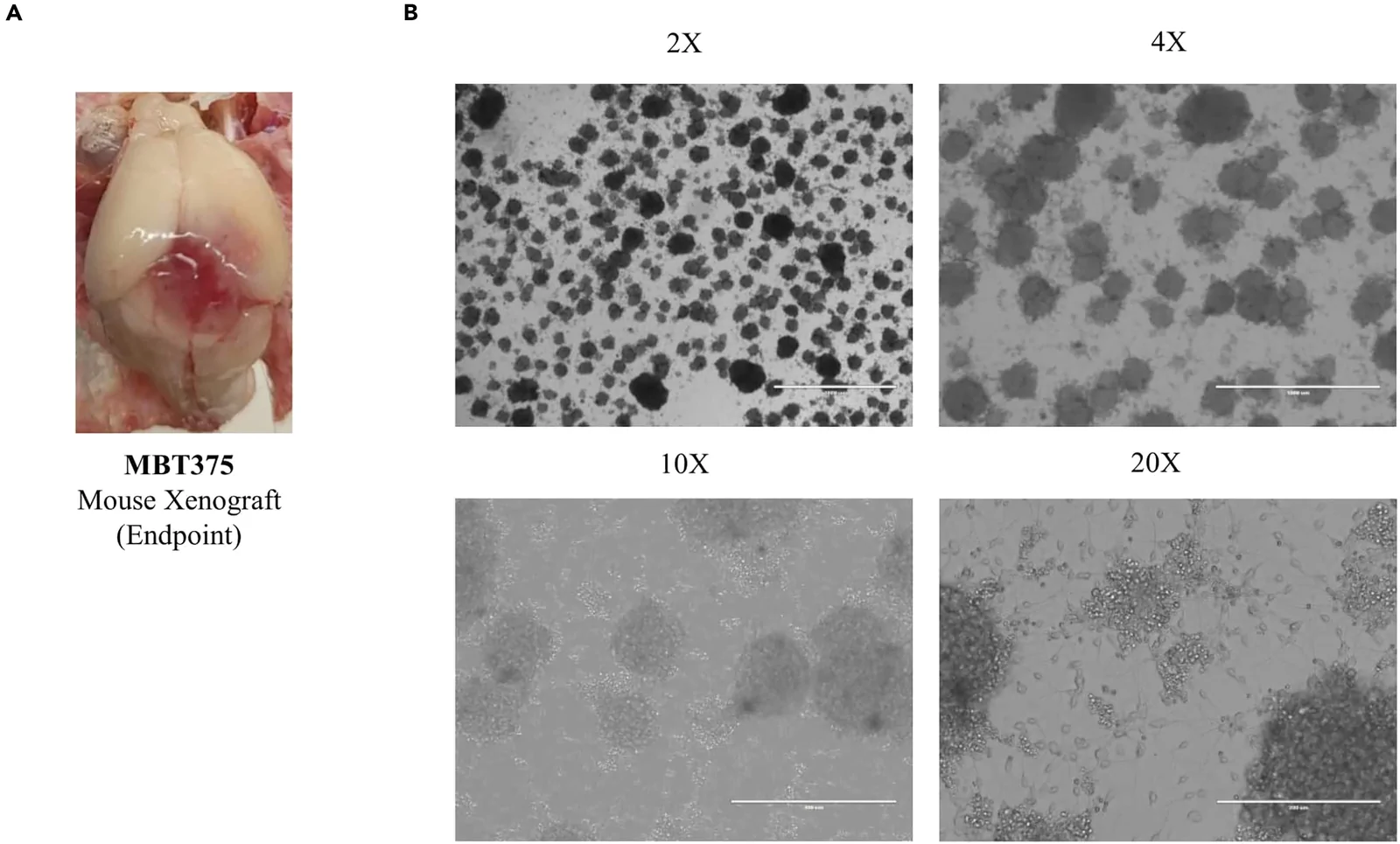
MBT375 tumor cell culture isolated from mouse brain tumor xenografts (A) Photographic image of a murine brain with a MBT375 PDX tumor at endpoint. (B) images of MBT37 tumor cell culture plated on POL taken after 24 h. MBT375 cells were isolated from a murine brain and cultured in NCC prior to plating on POL. Images were taken with an EVOS Cell Imaging System. Scale bars: 2X = 2000 m, 4X = 1000 m, 10X = 400 m, 20X = 200 m.

***Note:*** MBT375 brain tumors can be easily distinguished from healthy brain by their red and pink color. This phenotype greatly improves the excision of the tumor from healthy brain with razor blades (Figure 3A).

28. Isolating MBT375 tumor cells from mouse brain.
  a. Harvest mouse brain and collect it in 15 mL tube with ice cold PBS (on ice).
    ***Note:*** MBT375 tumors easily dislodge from mouse brain in PBS, simplifying the separation of mouse brain and MBT375 tumor cells.
  b. Place mouse brain and tumor in a 10 cm cell culturing dish and remove as much normal brain tissue from the tumor with razorblades as possible.
  c. Discard mouse brain in container with bleach.
  d. Collect all MBT375 from the 10 cm plate into 15 mL tube by ringing with PBS (max 10 mL).
  e. Dissociate MBT375 cell clumps into single cells by using a 16-gauge needle and 3 mL syringe to pipette PBS up and down 3–5 times.
    ***Note:*** MBT375 tumor cells easily dissociate into single cell suspension. The use of a 16-gauge needle for mechanical dissociation is sufficient, there is no need for additional enzymatic dissociation.
  f. Place a 70 μm filter on top of a 50 mL falcon tube.
  g. Remove residual mouse debris by pipetting the single cell suspension from the syringe on top of the 70 μm filter.
  h. Wash filter with 5 mL PBS to collect all tumor cells in the 50 mL falcon.
  i. Centrifuge filtered cell suspension at 430 × *g*, 10 min.
    **CRITICAL:** MBT375 tumor cells are very small in size. For this reason, we recommend that all centrifugation steps are performed at 430 × *g* for at least 10 min to ensure all single cells are pelleted.
  j. Discard supernatant by careful pipetting.
  k. Resuspend cell pellet in 1 mL warm NCC and count the number of viable cells with Trypan Blue and a cell counter.
29. Sphere formation Troubleshooting 3.
  a. Dilute Cell suspension to 10 mL with warm NCC and plate in a regular 10 cm culturing plate.
  b. Incubate plate in incubator for 24 h.
  c. Monitor plate for 2–4 days for sphere formation.
    ***Note:*** The culture will have a high number of cells that seem dead (debris-like phenotype) but they will recover, do not discard.
  d. When healthy spheres have formed, collect the spheres from the 10 cm plate into a 50 mL tube.
  e. Rinse the 10 cm plate with 10 mL PBS to ensure all tumor cells were collected.
  f. Centrifuge at 430 × *g*, 10 min.
    **CRITICAL:** MBT375 tumor cells are very small in size. For this reason, we recommend that all centrifugation steps are performed at 430 × *g* for at least 10 min to ensure all single cells are pelleted.
  g. Carefully discard supernatant by pipetting and carefully resuspend cells in 6 mL NCC.
  h. Plate cells onto 2 wells of a 6 well plate (POL-coated) in 3 mL each.
  i. Incubate plate in incubator for 24 h to allow cells to attach to POL and form a monolayer (Figure 3).
    **CRITICAL:** MBT375 cultures when stressed, such as isolation from mouse brains, may look dead like debris-like. Despite this phenotype, the cells will recover and form healthy cultures overtime. It is advised to be patient. Do not discard debris-like cultures at this stage.
  j. Maintain culture *in vitro* as described below.

### *In vitro* culturing: Passaging of MBT375 cells

**Timing: 1–2 h**

MBT375 cultures consists of two cell populations: MBT375 tumor cells and feeder cells.

**Figure 4:**
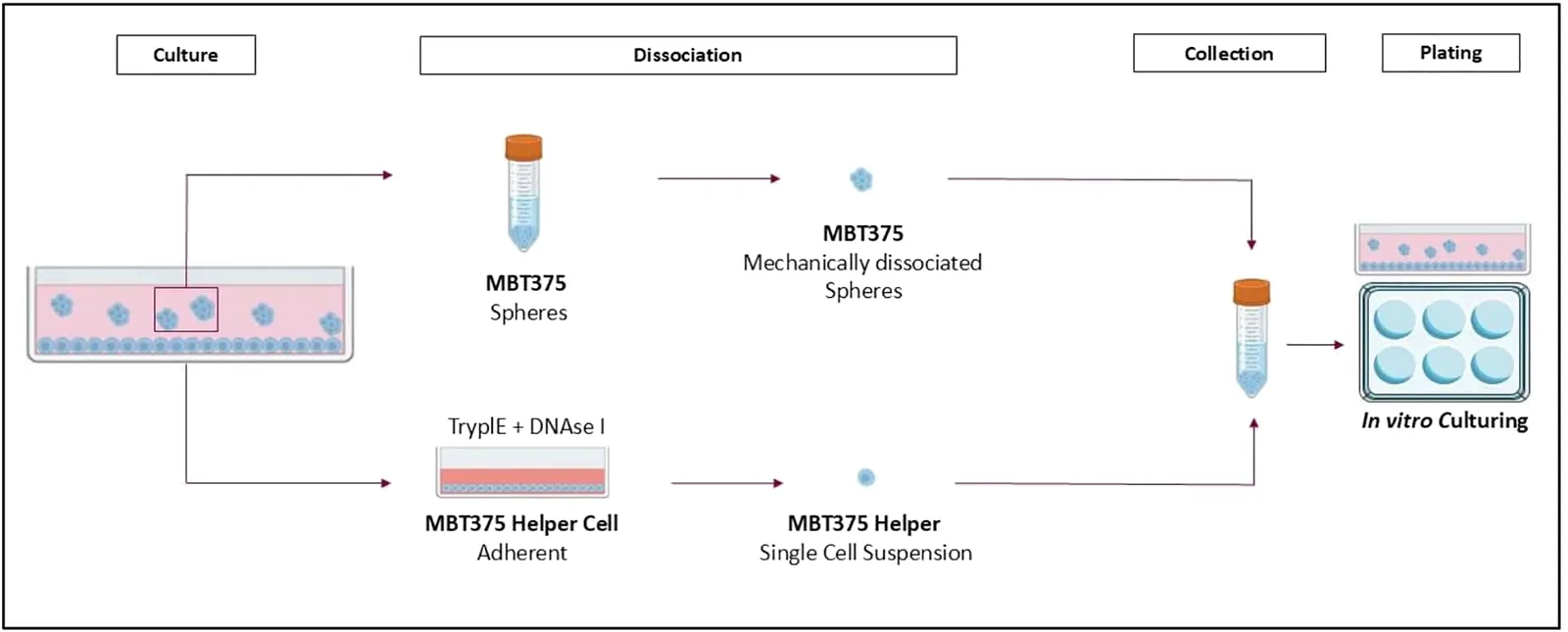
*In vitro* passaging of MBT375 spheres and feeder cells Schematic representation of the in vitro passaging protocol. Suspension tumor spheres are collected and mechanically dissociated into smaller spheres. The adherent monolayer of tumor helper cells is enzymatically dissociated. Both populations are combined in a new POL-coated plate.

Passaging of a MBT375 culture consists of dissociating tumor spheres into smaller spheres or single cells and enzymatically dissociating the feeder cell monolayer (Figure 4).

***Note:*** Passaging of MBT375 is dependent on 3 factors: sphere density, POL quality and media consumption.

30. Passaging of MBT375 suspension cells.
  a. Pre-warm NCC media in 37°C water bath.
  b. Remove media containing cells in suspension from culture plate and collect them in a falcon tube.
  c. Gently rinse off any left-over spheres from monolayer with 1 mL of PBS by using a P1000 pipette.
  d. Collect rinsing PBS in the same falcon tube with suspension MBT375 tumor cells.
  e. Proceed with Step 31 to process the adherent monolayer in parallel with the suspension cells.
  f. Centrifuge MBT375 suspension cells at 430 × *g* for 10 min.
  g. Discard supernatant from the suspension cell pellet and carefully dissociate MBT375 cells into smaller spheres by pipetting in 0.5 mL warm NCC.

***Note:*** It is possible that spheres need to be dissociated into smaller spheres prior to the feeder cell monolayer reaching confluency. In this case, the surplus number of spheres can be utilized in *in vitro* assays. Alternatively, these surplus spheres can be used to establish a new culture in a POL-coated 12 wp as well. Keep in mind that this culture might not last long *in vitro*.

31. Passaging of MBT375 adherent monolayer (feeder cells).
  a. Immediately after rinsing the MBT375 suspension cells of the plate (Step 30), add 500 μL of TrypLE + 10 DNAse I per 6 wp well to monolayer (10 μL of DNAse I per 500 μL TrypLE).
    ***Note:*** DNAse I is used to reduce debris aggregation during enzymatic dissociation.
  b. Incubate TryPLE plate at room temperature (20°C–25°C).
  c. When cells are detached from plate, rinse cells from plate with PBS and collect in Falcon tube.
  d. Rinse plate with PBS and collect cells in a Falcon tube. Repeat until all cells are collected.
  e. Centrifuge MBT375 cells at 430 × *g* for 10 min.
    **CRITICAL:** MBT375 tumor cells are very small in size. For this reason, we recommend that all centrifugation steps are performed at 430 × *g* for at least 10 min to ensure all single cells are pelleted.
  f. Resuspend in 0.5 mL warm NCC.
32. Combining MBT375 suspension and feeder cells Troubleshooting 4.
  a. Combine the 0.5 mL of MBT375 spheres with the 0.5 mL of mouse Feeder cells.
  b. Add 1 mL per 6 wp well (POL-coated) or 2–4 12 wp wells (POL-coated) according to cell numbers.
  c. Incubate in incubator for 1 to 2 h.
  d. Carefully top up the culture with 1 mL warm NCC.
  e. Incubate for 24 h in an incubator.
  f. Monitor cells daily and top up with 200 μL NCC or refresh 50% of media volume when appropriate.

### *In vitro* culturing: Seeding MBT375 cells for *in vitro* assays

**Timing: 2–3 h**

**Figure 5:**
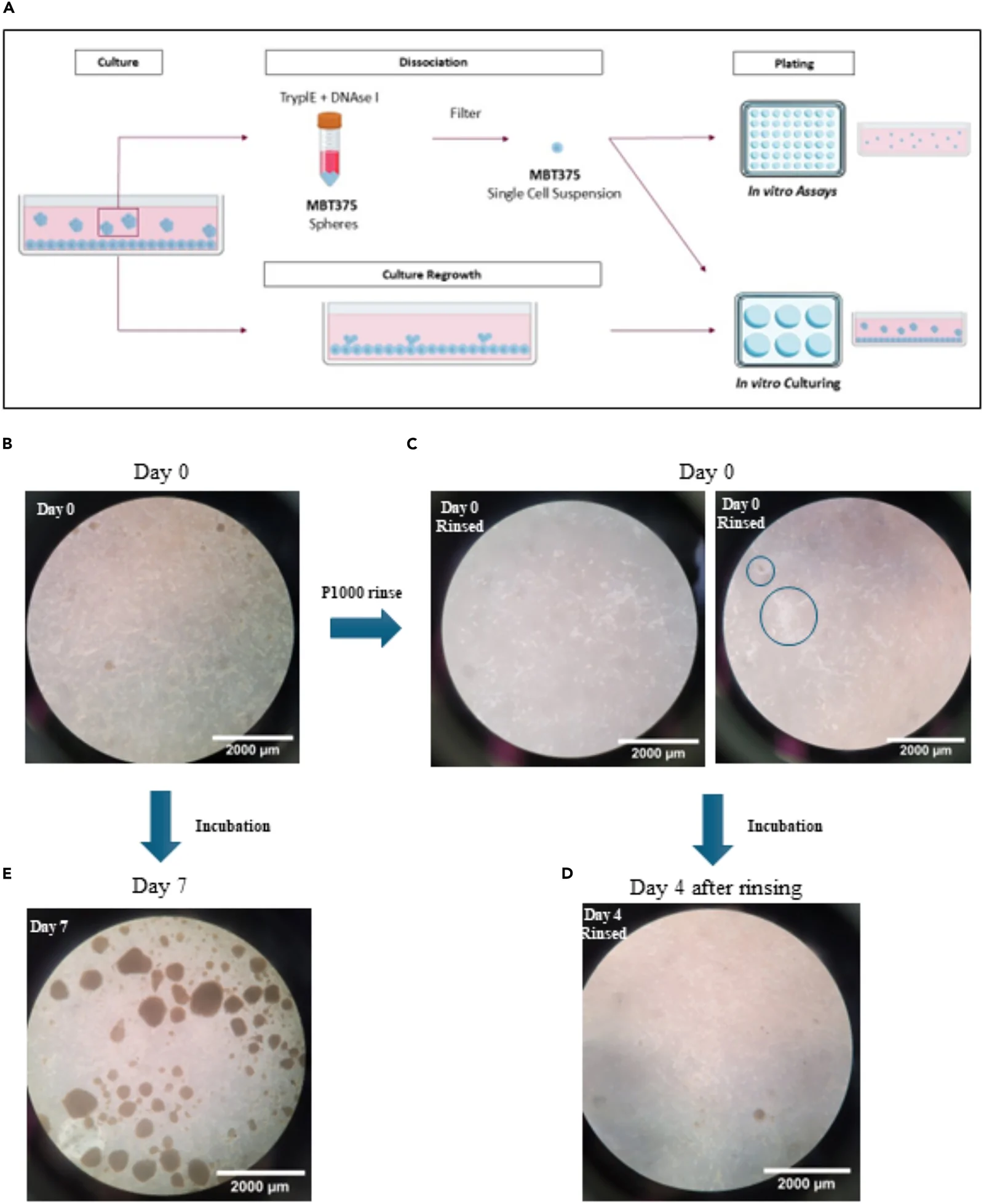
Seeding of MBT375 tumor cells *for in vitro* drug assays (A) Schematic representation of the *in vitro* assay seeding protocol. Tumor spheres in suspension are gently rinsed of the adherent monolayer of tumor helper cells and enzymatically dissociated into single cells with TryplE. DNAse I is used to reduce debris aggregation during enzymatic dissociation. The dissociated single tumor cells are seeded in assays while the main culture is allowed to regrow. (B–E) Microscope images of a stable culture at day 0 and day 7 (without pipetting intervention). (B–D) Microscope images of a stable culture during the collection of tumor spheres for in vitro assays. (B) Stable MBT375 population cultured on a POL-coated plate in NCC media. (C) adherent MBT375 cells (B) after collection of suspension cells by gentle PBS rinsing with P1000 pipet. (D) MBT375 culture (C) four days after suspension cell collection. (E) MBT375 culture left to grow for 7 days, with highly dense spheres in need of dissociation. Scale bars are 2000 m.

The protocol for seeding MBT375 cells for assays slightly deviates from the passaging protocol as the feeder cell monolayer is not dissociated. Suspension spheres are collected and enzymatically dissociated into single cells. (Figure 5).

***Note:*** Surplus cells not required for the assay can be reseeded into its original plate or seeded atop another MBT375 culture.

33. Collecting MBT375 suspension spheres.
  a. Pre-warm NCC media in 37°C waterbath.
  b. Remove media containing suspension cells from plate and collect in a falcon tube.
  c. Gently rinse off any left-over spheres from monolayer with 1 mL of PBS by using a P1000 pipette.
  d. Collect the monolayer cells in the same falcon tube with suspension MBT375 cells.
  e. Centrifuge MBT375 cells at 430 × *g*, 10 min and add 2 mL of warm NCC to the monolayer for culturing.
  f. Discard supernatant.
34. Enzymatic dissociation of spheres into single cells Troubleshooting 5.
  a. Resuspend MBT375 spheres in 500 μL TrypLE + 10 μL DNAse I.
    ***Note:*** MBT375 cells are sensitive to enzymatic dissociation. Extracellular DNA will induce formation of agglomerate precipitated debris. These agglomerates should immediately remove by pipetting to limit loss of cells.
  b. Incubate at room temperature (20°C–25°C) and actively resuspend by using a P200 pipette.
    ***Note:*** Spheres tend to stick to the falcon tube, ensure all spheres are in suspension inbetween pipetting.
  c. Monitor the resuspended solution under a light microscope to ensure single cell suspension is achieved.
  d. Dilute single cells with 5 mL of warm NCC.
  e. Centrifuge at 430 × *g* for 10 min.
    **CRITICAL:** MBT375 tumor cells are very small in size. For this reason, we recommend that all centrifugation steps are performed at 430 × *g* for at least 10 min to ensure all single cells are pelleted.
35. Seeding cells for *in vitro* drug assays.
  a. Resuspend single cells in 500 μL NCC.
  b. Count cells in duplicate.
  c. Plate MBT375 cells in a flat bottom 96 well plate (approximately 10,000 - 30,000 cells per well).
    ***Note:*** Due to the small MBT375 cell size and relatively low metabolic activity, the use of 10,000 - 30,000 cells per 96 wp well is recommended to ensure reliable *in vitro* viability assay readouts.
  d. Incubate cells for 24 h in an incubator before starting an *in vitro* assay.
  e. Reseed any surplus cells into a low binding plate.
  f. Incubate for 24–48 h until spheres have formed.
  g. Plate newly formed spheres into the parent MBT375 culture.
    ***Note:*** MBT375 cells that have been dissociated into single cells may have difficulty reforming into spheres, especially with low cell numbers. The cell repellent properties of low binding plates aids in cell recovery and sphere formation as it forced the cells into close proximity.
    ***Note:*** Prior to using a MBT375 population isolated from a mouse brain, flow cytometry using human Tra1 antibodies can be considered to determine the percentage of mouse cells. The use of mouse cell depletion kits can be considered prior to drug assay seeding.

### *In vitro* culturing: Multiple assay workflow

**Timing: 1–3 months**

**Figure 6:**
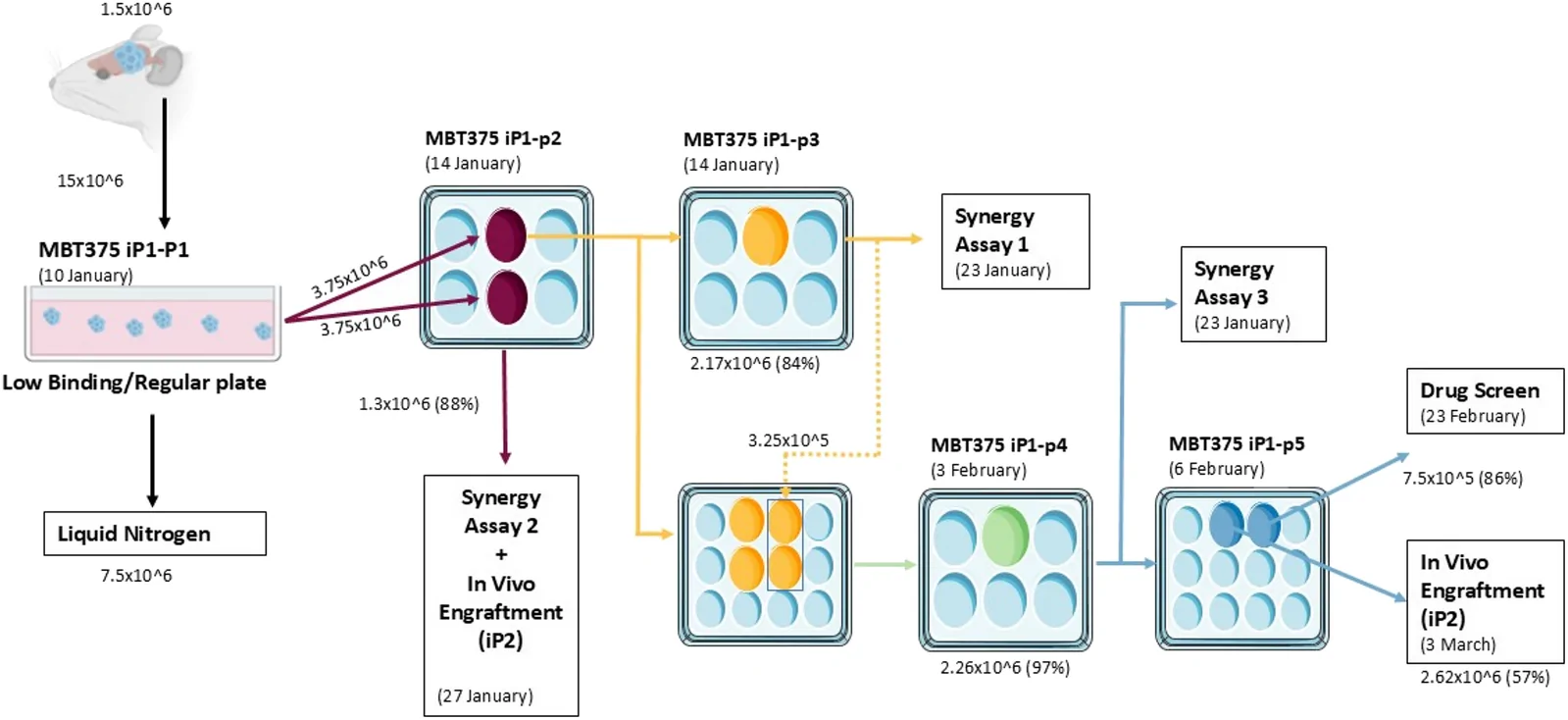
Optimized culturing strategy to maximally utilize a MBT375 culture for multiple *in vitro* assays Schematic representation of the workflow of a MBT375 culture isolated from a murine PDX tumor, cultured for three passages while collecting cells for subsequent murine-serial engraftment, cryopreservation and multiple in vitro assays. During culturing different well sizes are utilized for optimal cell density.

In order to maximally utilize a single *in vitro* culture and perform multiple assays, we here describe a workflow from brain isolation to multiple *in vitro* passages, combined with *in vitro* assays and serial engraftments to ensure optimal use of MBT375 cells *in vitro* (Figure 6).

***Note:*** The low proliferative capacity does not permit sufficient growth for bulk *in vitro* expansion. The subtraction of cells from a culture for *in vitro* assays therefore reduces a cultures ability to re-establish itself.

36. Inject MBT375 cells into mice and start endpoint monitoring.
37. Upon endpoint collect whole mouse brains and isolate MBT375 cells.
38. Establish an *in vitro* culture.
39. When culture is ready for passaging, cryo-preserve half of the culture.
40. Plate the other half of the culture onto 2 wells of a POL-coated 6 wp.
41. When the 2 × 6 wp well cultures are ready to be passaged, serial engraft 1 × 6 wp culture *in vivo* and seed half of the other 1 well/6 wp (POL-coated) well culture for an in vitro assay and replate the other half into a new 6 wp well (POL-coated), or 4 wells/12 wp (POL coated) if culture does not look healthy. Troubleshooting 6.
42. Combine 4 wells/12 wp (POL-coated) culture onto 1 well/6 wp (POL-coated) when ready to split.
43. Continue the process seeding half of a 6 wp well (POL coated) culture for *in vitro* assays and passaging the other half into a new 6 wp (POL-coated).
44. When *in vitro* cultures start having trouble to recover after passaging, immediately serial engraft *in vivo.*

## Expected outcomes

Our protocol generates G4-MB models that retain G4-MB characteristics *in vitro* and after serial murine orthotopic engraftment.^1^ Using this protocol, data can be derived from *in vitro* drug assays in a reproducible manner, hereby overcoming a major bottleneck for pre-clinical development. Keep in mind that these tumor cells are fragile *in vitro* and handling these tumor cells requires a certain finesse that will be developed over time. Serial-engraftment in mice will increase robustness of tumor cells and improve *in vitro* performance.

G4-MB tumor cells will form spheres when cultured in NCC media and will aggregate into spheroid clusters. When plating these spheroid structures on Poly-L-Ornithine + laminin (POL) coated plates, a supportive adherent layer of feeder cells will form onto which spheres will tether.

Orthotopic engraftment of single cell dissociated G4-MB tumor cells in mice can have varying success rates ranging from approximately 40%–60%. Directly injecting small spheres improves engraftment rates and may be preferred when trying to expand MBT375 tumor cell stocks. Upon endpoint these tumors can reach a mass of a maximum 20–50 million cell per tumor. These tumors have a characteristic red color which easily detach from the brain.

Importantly, when mice approach endpoint and start to lose weight, they remain stable for relatively long when compared to more aggressive MB models such as MYC amplified G3-MB. Mice bearing G4-MB tumors can remain stable for about 1 week when clinical symptoms manifest.

When cultured *in vitro*, MBT375 tumor cells can be used to generate reproducible assay results when used as described in this protocol.

## Limitations

Even though this protocol is designed for the *in vitro* culturing of patient-derived G4-MB tumor cells, the number of passages is limited. The low proliferation rate of MBT375 cells *in vitro* means splitting depletes the ability to re-establish a healthy culture, especially when re-allocating cultured cells to assays.

Variability in MBT375 tumor cell and feeder cell proportions may arise between tumor xenograft derived cultures, which may impact proliferative capacity and sphere forming capacity. When tumor cell numbers are too low, effective sphere formation is reduced, and the culture may not yield sufficient spheroids for study.

Bulk propagation of G4-MB cells mainly relies on serial engraftment in mice. Upon endpoint this protocol can be utilized to conduct *in vitro* studies. Another potential limitation is the engraftment duration. Xenograft tumors require a period of approximately 30 days to stabilize prior to exponential growth. During the exponential growth phase endpoint is reached after 9–12 months.

This protocol is designed for short-term manipulation of patient-derived MBT375 tumor cells while preserving G4-MB biological identity within the timeframe demonstrated in the original publication. When using the model for longer-duration, re-evaluation of tumor identity is advised.

## Troubleshooting

### Problem 1

No live cells are detected during counting cells (Related to all sections).

### Potential solution

• Be sure to manually adjust the Cell Counter focus area to ensure round cells are in view. MBT375 cells have very small cell bodies and appear as debris after Cell Counter autofocusing, resulting in low viability counts.
• Disregard these low viability counts when focus area adjustment does not yield higher viable cell counts. Do not discard the cells yet, as they most likely recover over time with proper care. MBT375 cells display a debris-like phenotype when they are stressed (e.g., thawing from liquid nitrogen and when freshly isolated from mouse brains).
• Be sure to pipet gently pipetting, refrain from using p1000 pipettes as rigorous use of P1000 pipettes may induce cell death.
• MBT375 cells do not withstand the shear stress during FACS sorting. Sorted MBT375 cells are not be viable to start a new culture.
• Manual counting by using a Neubauer chamber can also be considered as an alternative to accommodate for potential Cell Counter errors.

### Problem 2

Low engraftment success in mice (“intracranial engraftment in mice” section).

### Potential solution

• Refrain from injecting cells with a stressed debris-like phenotype as they are less likely to survive the injection/engraftment process. Allow cells to recover in low binding plates with NCC media to ensure healthy sphere formation.
• Be sure to pipette gently, refrain from using p1000 pipettes.
• Increase cell numbers per mouse to overcome engraftment limitations.
• When unable to generate successful xenografts, refrain from injecting single cells. Often overhandling can limit engraftment potential. Alternatively, collect mechanically dissociated spheres in PBS and gently resuspend in appropriate injection volume without dissociating into single cells. Distribute the spheroid cells by intracranially injecting them into a cohort of mice (minimum of 5 mice per 10 cm dish culture).
• Engraftment rates can vary when injecting single cells. When accurate cell numbers are not required, e.g., when replenishing tumor cell stocks *via* serial engraftment, injecting intact small spheres after mechanical dissociation is preferred to ensure proper engraftment.

### Problem 3

Cultures seeded after patient tumor or mouse brain isolation look mostly like debris (“G4-MB patient tumor processing” and “mouse PDX tumor isolation” sections).

### Potential solution

• Do not discard the cells, give them approximately 1 week to recover. Over time, cells should aggregate and form debris-like clouds and subsequently start forming sphere like aggregates. At this stage these aggregates should be gently plated onto POL-coated plates in a low volume of NCC and incubated for 24–48 h to allow culture stabilization onto the POL.
• When culture does not recover after 1 week, transfer the cells into a smaller plate, or use a low binding plate prior to seeing onto a POL-coated plate.

### Problem 4

Culture does not adhere properly to POL. The cells are not attached to the POL coated plate when seeding or large patches of adherent cells are lifting off the plate surface in an established culture (“in vitro culturing: Passaging of MBT375 cells” section).

### Potential solution

• Ensure adequate volumes of Poly-L-Ornithine and Laminin are used while preparing POL-coated plates. When volumes are too low, the outer edges of the plate surface will not be submerged, preventing a fully coated plate surface from forming. From these uncoated sections cracks in the POL will form overtime, resulting in sheets of POL tearing off the plate surface over time.
• POL-coated cell culturing plates last for approximately 2 weeks at 37°C before their quality starts to diminish. When POL quality diminishes sections of POL coating will detach for the plate or sections will be pulled together under a sphere tethered to monolayer, leaving patches of empty space (Figure 5).

### Problem 5

Excessive cell death occurring during enzymatic dissociation, resulting in the formation growing sticky precipitated masses of debris (“in vitro culturing: Seeding MBT375 cells for in vitro assays” section).

### Potential solution

• To minimize cell death, TrypLE (TE) is used and mixed with DNAse-I. During enzymatic dissociation extra cellular DNA will form aggregates with debris and cells, the addition of DNAse-I helps minimize this aggregate accumulation. Enzymatic dissociation should always be performed at room temperature (RT) to minimize aggregate accumulation.
• Gently resuspend with a P200 pipet to speed up the process (be careful keep live spheres in solution as they tend to stick to the falcon tube wall). Keep in mind that in these conditions extracellular DNA/debris conglomerates can still arise. Always monitor the cell suspension under a microscope until all cells have been dissociated to single cell and immediately remove any DNA/debris conglomerates to prevent cell loss.
• Excessive precipitated debris aggregate formation is a sign that spheres were too dense and should have been split earlier. When spheres become too dense cell death occurs in the core due to nutrient and oxygen deprivation. When sphere density increases the sphere turns dark brown to black when viewed under a light microscope. Spheres should be passaged before reaching this dark brown colored phenotype.
• Media conditions were too acidic for cells to survive. Fresh NCC media has a pink/orange color which turns yellow in acidic conditions. Ensure media color remains in the pink-orange range. When top ups are not sufficient to keep media color in the recommended range, remove 50% of media volume and top-up the culture with an equal volume of fresh media. Always retrieve any potential cells in by centrifugation and resuspend the cells in fresh top-up media.

### Problem 6

Cell culture does not stabilize into a monolayer with healthy (“in vitro culturing: Multiple assay workflow” section).

### Potential solution

• MBT375 cell cultures are highly sensitive to the ratio of cells to surface area, and cultures will not fully mature when seeded too sparsely. Seed higher density of cells to prevent this from occurring.
• When cell numbers are limited, the use of 6 well plates (6 wp) and 12 well plates (12 wp) are recommended. Seed 2–3 million cells per 6wp well. When a 6 wp culture is struggling, transfer the culture into smaller sized wells (e.g., 4 wells of a 12 wp). When the 12 wp cells are healthy, they can be recombined into a 6 wp well.
• Reduce media volume when seeding cells to stimulate binding of cells to POL.
• Pre-incubate cells in low binding plates for 24–48 h to stimulate sphere formation prior to seeding onto POL.

## Resource availability

### Lead contact

Further information and request for resources and reagents should be directed to and will be fulfilled by the lead contact, Sheila K. Singh (ssingh@mcmaster.ca).

### Technical contact

Technical questions regarding the execution of this protocol should be directed to and will be answered by the technical contact, Stefan Custers (custerss@mcmaster.ca).

### Materials availability

For requests of biological materials, please refer to the lab director, Chitra Venugopal (venugop@mcmaster.ca).

### Data and code availability

This study does not report any datasets or computer codes.

## Acknowledgments

We are grateful to the patients who donated tumor tissue for this project. This study was supported by grants from the Canadian Institutes of Health Research (10.13039/100022992CIHR, no. 400498); the Ontario Institute for Cancer Research (10.13039/100012118OICR) Cancer Stem Cell Program; the Canadian Cancer Society Research Institute (10.13039/501100000015CCSRI, no. 707420); and donations from the Box Run Foundation and Team Kelsey Foundation. This work greatly benefited from the support of the Medulloblastoma Initiative (MBI) and the involvement of the affiliated Cure Group Four Consortium (CGFC). Special thanks go to the Goldsztein Family for supporting this research. This work is dedicated to the MBI patient. The graphical abstract and figures were partially created using Biorender.com.

## Author contributions

S.K.S., C.V., and S.C. conceived and designed this study. S.C. performed the experiments and wrote the manuscript. S.K.S. and C.V. supervised the project and edited the manuscript.

## Declaration of interests

The authors declare no competing interests.
